# Adult Current Smoking: Differences in Definitions and Prevalence Estimates—NHIS and NSDUH, 2008

**DOI:** 10.1155/2012/918368

**Published:** 2012-05-09

**Authors:** Heather Ryan, Angela Trosclair, Joe Gfroerer

**Affiliations:** ^1^Division of Cancer Prevention and Control, Centers for Disease Control and Prevention, National Center for Chronic Disease Prevention and Health Promotion, Atlanta, GA 30341, USA; ^2^Office on Smoking and Health, Centers for Disease Control and Prevention, National Center for Chronic Disease Prevention and Health Promotion, Atlanta, GA 30341, USA; ^3^Substance Abuse and Mental Health Services Administration, Center for Behavioral Health Statistics and Quality, Rockville, MD 20857, USA

## Abstract

*Objectives*. To compare prevalence estimates and assess issues related to the measurement of adult cigarette smoking in the National Health Interview Survey (NHIS) and the National Survey on Drug Use and Health (NSDUH). *Methods*. 2008 data on current cigarette smoking and current daily cigarette smoking among adults ≥18 years were compared. The standard NHIS current smoking definition, which screens for lifetime smoking ≥100 cigarettes, was used. For NSDUH, both the standard current smoking definition, which does not screen, and a modified definition applying the NHIS current smoking definition (i.e., with screen) were used. *Results*. NSDUH consistently yielded higher current cigarette smoking estimates than NHIS and lower daily smoking estimates. However, with use of the modified NSDUH current smoking definition, a notable number of subpopulation estimates became comparable between surveys. Younger adults and racial/ethnic minorities were most impacted by the lifetime smoking screen, with Hispanics being the most sensitive to differences in smoking variable definitions among all subgroups. *Conclusions*. Differences in current cigarette smoking definitions appear to have a greater impact on smoking estimates in some sub-populations than others. Survey mode differences may also limit intersurvey comparisons and trend analyses. Investigators are cautioned to use data most appropriate for their specific research questions.

## 1. Introduction

Cigarette smoking continues to be the single greatest preventable cause of disease and death in the United States [1]. The US federal government's first nationally-representative survey of cigarette smoking and other tobacco use behaviors took place in 1955 as a supplement to the US Census [2]. Since then federally sponsored tobacco surveillance has grown to include several established data collection systems routinely implemented at the national level, some of which have been adapted, sponsored, and implemented at the state level [3–5]. As one of the World Health Organization (WHO) MPOWER package's six proven tobacco prevention and control policies [6], tobacco prevention and control monitoring systems and their maintenance and enhancement are an essential part of public health practice [7]. Specifically, WHO calls for monitoring systems that track multiple anti- and protobacco attitude, behavior, and policy indicators; disseminate findings to facilitate utilization; provide overall as well as demographic subpopulation data at the national, state, and, where practicable, local levels; maximize system sustainability through cross-discipline collaboration, strong management and organization, and sound funding [6].

Understanding, documenting, and quantifying the characteristics of the tobacco user, or potential user, have been key to tobacco control efforts [4]. A variety of existing monitoring, research, and evaluation systems are available to collect such information [4], with increasing demand for surveillance data to inform evidence-based public health tobacco initiatives necessitating their periodic review [5]. At the national level, the National Health Interview Survey (NHIS) has been the data source used to measure progress on Healthy People adult tobacco-use prevalence objectives since the first ever release of national health objectives (Healthy People 1990) [8, 9]. Adult tobacco-use prevalence can be estimated from other national surveys as well [3], allowing evaluation of any differences in prevalence magnitude or in trends over time between data sources; however, there have been few studies comparing their smoking prevalence estimates [10]. A comparison of estimates from the 1997 NHIS and national estimates from the 1997 Behavioral Risk Factor Surveillance System (BRFSS) surveys [11] found current smoking prevalence to be significantly higher in NHIS than in BRFSS (24.7% versus 23.1%). Differences were also observed in a Substance Abuse and Mental Health Services Administration (SAMHSA) report [12] that described smoking prevalence estimates from the 2005 National Survey on Drug Use and Health (NSDUH). SAMHSA reported that estimates from NSDUH were higher (26.5%) than estimates obtained from the 2005 NHIS (20.9%), even after applying the NHIS current smoking definition to NSDUH data limiting smokers only to those who reported smoking ≥100 cigarettes in their lifetime (24.7% in NSDUH using NHIS definition). In a 2009 report comparing NHIS and NSDUH current smoking prevalence for the period 1998–2005, Rodu and Cole [10] describe an increasingly divergent picture of smoking prevalence in the USA between 1999 and 2005. Rodu's secondary analysis of NHIS and NSDUH data indicated that by 2005 NHIS prevalence had declined to approximately 21% while the NSDUH estimate was approximately 25%, with the latter but not the former suggesting a plateau in smoking prevalence. This pattern then reversed with a 2010 report using NHIS data that indicated a stall in the prevalence of adult smoking from 2005 (20.9%) to 2009 (20.6%) [13] while SAMHSA's primary analysis of NSDUH data suggested a continuing decline from 26.5% to 24.9% during the same period [12].

Key methodological issues, such as sampling design, survey mode and setting, and survey question standardization and context, have the potential to influence data quality and comparability [4]. Differences in the survey questions used to define current smoking are thought to be one of the probable methodological sources of discrepancy between NHIS and NSDUH smoking estimates. Most notably, NHIS limits its question of current smoking to respondents who on a previous question reported smoking ≥100 cigarettes in their lifetime (i.e., NHIS “ever smokers,” with “never smokers” then defined as respondents with lifetime smoking anywhere between 0 and 99 cigarettes). NSDUH also limits its current smoking definition based on reported ever smoking behavior; however, other than an implicit zero, it does not designate a cut-point for number of lifetime cigarettes smoked for categorizing “ever smokers” versus “never smokers.”

Levels of cigarette consumption—such as number of cigarettes smoked per day, number of days smoked per month, and amount of lifetime cigarette use—have often served as a proxy for other key tobacco control indicators, such as secondhand smoke exposure, nicotine addiction, and health risk [14]. This, however, may not necessarily be advisable practice. A review by Husten (2009) [14] concluded that consumption is a crude measure of both toxin exposure and nicotine dependence and, with respect to toxin exposure, likely inaccurate as well. Likewise, with respect to health risk, the review concluded that no level of consumption could be considered “safe,” and thus used to demarcate a risk threshold. Research specific to whether 100 lifetime cigarettes is a discriminating cut-point for distinguishing ever smokers versus never smokers—and, subsequently, for defining who is, ever has been, or may become a current smoker—is limited [15] but indicates that it too may be unsuitable. In a study of craving patterns, tolerance, and subjective responses to the pharmacological effects of smoking, findings from Pomerleau et al. (2004) [16] indicated 20 cigarettes per lifetime may be a more prudent marker than 100 for such a differentiation. Others have proposed that liability for dependence and subsequent uptake of smoking may even be distinguishable after an individual's very first puff [17]. Additionally, non-daily and light daily smoking—behaviors consistent with current cigarette smoking but lifetime smoking <100 cigarettes—have been found to significantly vary across racial/ethnic subpopulations [18–24]. Findings from Trinidad et al. (2009) [24] indicated non-Hispanic black, Asian/Pacific Islander, and Hispanic/Latino smokers were more likely to be nondaily and light daily smokers compared with non-Hispanic whites, even after controlling for age, gender, and education level. This was particularly true of Hispanic/Latino smokers, who were 3.2 times more likely to be non-daily smokers and 4.6 times more likely to be daily smokers who smoke ≤5 cigarettes per day as compared with non-Hispanic white smokers. Furthermore, Hispanic/Latino non-daily smokers smoked fewer days per month and smoked fewer cigarettes per day on the days they did smoke compared with non-Hispanic whites.

Infrequent smoking and smoking trajectories among adults remain open research issues. Youth data emerging over the past decade, however, have consistently concluded the trajectory of smoking begins with the loss of autonomy that occurs during infrequent use [25–30]. Among adults who have adopted the practice of infrequent smoking, research not only suggests it can remain a stable pattern lasting long periods of time [31–33] but that it also poses substantial health risk with adverse outcomes paralleling dangers observed among daily smoking, especially for cardiovascular disease [34]. Such results have notable implications for the understanding of tobacco dependence and the development of prevention and cessation strategies, especially for racial/ethnic minorities.

While differences in current smoking estimates between NHIS and NSDUH have been previously reported [10, 12], more in-depth examination directed specifically at methodology and how differences may affect comparability with other surveys is needed [10, 35]. Therefore, the current report makes comparisons between NHIS and NSDUH prevalence estimates using, for NHIS data, the standard NHIS definition of current smoking, which includes a screener question for a level of lifetime smoking ≥100 cigarettes and, for NSDUH data, using both the standard NSDUH definition of current smoking, which does not use the screener question, and a modified definition that applies the NHIS current smoking definition (i.e., with 100-cigarette restriction) to NSDUH data. Specifically, the following research questions are addressed: (1) how and for what subpopulations and smoking behaviors might the ≥100 lifetime cigarettes criterion affect adult prevalence estimates? and (2) what subpopulations are most likely to have smoked during the past 30 days but not meet the ≥100 lifetime cigarettes criterion? Findings are presented by sociodemographic characteristics for current smoking and for daily smoking among current smokers.

## 2. Materials and Methods

### 2.1. Surveys

We used data from the 2008 NHIS and 2008 NSDUH public data files for prevalence comparisons between surveys. Combined 2006–2008 NSDUH public data files were used to examine subpopulation characteristics of respondents who had smoked during the past 30 days but did not meet the ≥100 lifetime cigarettes criterion.

### 2.2. NHIS

The NHIS is a multipurpose national health survey conducted by the National Center for Health Statistics (NCHS) at the Centers for Disease Control and Prevention (CDC) and is designed to provide information about a wide range of health topics for the noninstitutionalized US household population aged 18 years and older. The survey uses multistage, cluster sampling. It is primarily administered as a direct in-person interview, with interviews that either cannot be conducted or fully completed in person administered by telephone. The percentage of completed 2008 NHIS sample adult interviews that were administered either in part or in whole by telephone was 25% (S. Jack, NCHS, personal communication, Oct. 19, 2011). Interviews are conducted by field representatives using computer-assisted personal interviewing (CAPI). The CAPI data collection method employs computer software that presents the questionnaire on a computer screen and guides the interviewer through the questionnaire, automatically routing them to appropriate questions based on answers to previous questions. Interviewers enter survey responses directly into the computer, and the CAPI program determines if the selected response is within an allowable range, checks it for consistency against other data collected during the interview, and saves the responses into a survey data file. The nationally representative survey sample and subsequent data weighting permit calculation of national estimates. In 2008, the design oversampled non-Hispanic black, Hispanic, and Asian populations to allow for more precise estimates in these groups. The 2008 household response rate was 84.9%, and the interview response rate was 74.2%, yielding an overall response rate of 62.9%. Further details about the sampling and survey methodology used in the NHIS can be found elsewhere [36].

### 2.3. NSDUH

The NSDUH is a national health survey sponsored by SAMHSA and is designed to provide information about the use of alcohol, tobacco, and illegal drugs in the non-institutionalized US household population aged 12 years and older [37]. The survey sample design is a stratified, multistage, area probability design. Since 1999, the survey has been administered through confidential, anonymous, face-to-face interviews in the household by trained interviewers using a combination of direct CAPI and audio computer-assisted self-interviewing (ACASI) in which the respondent reads questions on a computer screen or listens to questions through headphones and then records answers into a computer, to increase honest reporting of sensitive behaviors. The tobacco-use section was conducted via self-administered ACASI. The representative survey sample and subsequent data weighting permit calculation of national estimates. The design oversamples youth and young adults to allow for more precise estimates in these groups. There is no oversampling of racial/ethnic groups. The 2006 household response rate was 90.6%, and the interview response rate for adults ≥18 years [38] was 72.9%, yielding an adult overall response rate of 66.0%. The household, adult interview [39], and adult overall response rates were 89.5%, 72.7%, and 65.0%, respectively, for the 2007 survey and 89.0%, 73.3%, and 65.3%, respectively, for the 2008 survey. Further details about the sampling and survey methodology used in the NSDUH can be found elsewhere [37, 40, 41].

### 2.4. Variable Definitions

For both NHIS and NSDUH, we examined current smoking status and, among current smokers, daily smoking. For NSDUH, we also examined level of lifetime cigarette use among current smokers. Definitions for each measure follow.

### 2.5. Current Smoking

#### 2.5.1. NHIS

**Figure 1:**
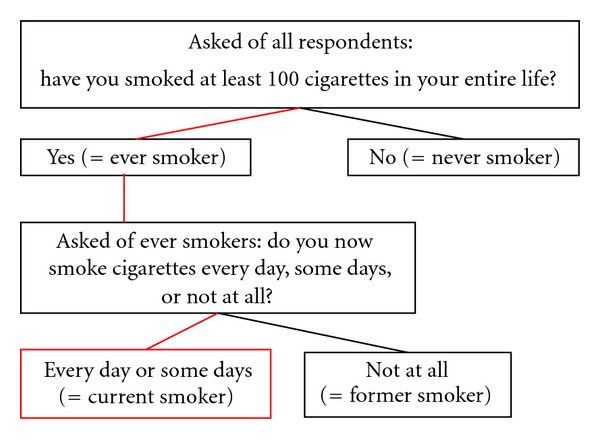
Standard NHIS current cigarette smoking variable definition.

The standard NHIS current smoking definition (hereafter simply termed the “NHIS definition”) has comprised of two questions [42] since 1965 (J. Madans, NCHS, personal communication, Nov. 10, 2011), with the present wording in use since 1992 [43]. The first question, asked of all respondents, is “have you smoked at least 100 cigarettes in your entire life?” Respondents answering “yes” are classified as ever smokers, and those who answer “no” are classified as never smokers and excluded from subsequent cigarette use questions. Ever smokers are then asked a second question: “do you now smoke cigarettes every day, some days or not at all?” Respondents who answer “every day” or “some days” are classified as current smokers (Figure 1).

#### 2.5.2. NSDUH

**Figure 2:**
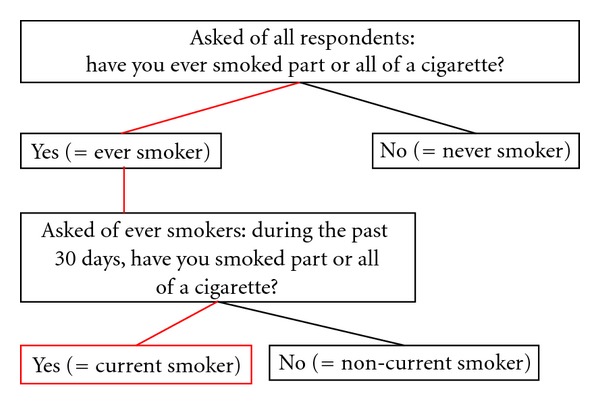
Standard NSDUH current cigarette smoking variable definition (NSDUH-S).

Our analysis used two different definitions of current smoking for NSDUH: the standard current smoking definition (NSDUH-S) established in 1993 and a modified definition (NSDUH-M) constructed to be comparable to the NHIS definition. The NSDUH-S current smoking definition uses two questions to measure smoking prevalence [44]. The first, asked of all respondents, is “have you ever smoked part or all of a cigarette?” Respondents answering “yes” are classified as ever smokers, and those who answer “no” are classified as never smokers. Ever smokers are then asked a second question: “during the past 30 days, have you smoked part or all of a cigarette?” Respondents who answer “yes” are classified as current smokers (Figure 2).

**Figure 3:**
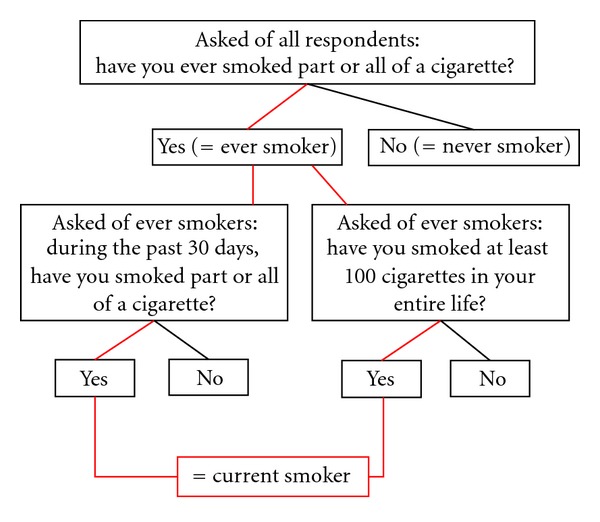
Modified NSDUH current cigarette smoking variable definition (NSDUH-M).

While NSDUH also contains the question “have you smoked at least 100 cigarettes in your entire life?” identical to the NHIS and is asked of NSDUH ever smokers, it is not used to define current smoking. We constructed the second, modified NSDUH-M current smoking definition that includes the 100-cigarette lifetime use question, with NSDUH-M current smokers defined as NSDUH ever smokers who both reported smoking part or all of a cigarette during the 30 days preceding the survey and reported lifetime cigarette use ≥100 cigarettes (Figure 3).

### 2.6. Daily Smoking

For NHIS, daily smoking among current smokers was defined primarily using the question “do you now smoke cigarettes every day, some days, or not at all?”, and secondarily using the question “on how many of the past 30 days did you smoke a cigarette?” which is asked of “some day” smokers only. Respondents who answered “every day” to the first question were classified as daily smokers, as were respondents who answered “some days” to the first question but for the second reported smoking a cigarette on all of the preceding 30 days. For NSDUH-S and NSDUH-M, this variable was defined using the question “during the past 30 days, that is, since [DATE], on how many days did you smoke part or all of a cigarette?” Respondents who answered that they smoked on all of the preceding 30 days were classified as daily smokers.

### 2.7. Lifetime Cigarette Use

For NSDUH-S, level of lifetime cigarette use among current smokers was defined using the question “have you smoked at least 100 cigarettes in your entire life?”, with dichotomized “yes/no” response options differentiating those who have smoked ≥100 cigarettes in their lifetime versus those who have smoked <100.

### 2.8. Demographic Information

For both surveys, smoking status was examined by age group (18–25, 26–34, 35–49, 50–64, ≥65), gender (male, female), race/ethnicity (non-Hispanic white, Non-Hispanic black, Hispanic or Latino, Asian, American Indian/Alaska Native), and education among persons aged ≥26 years (< high school, high school graduate, some college, college graduate).

### 2.9. Statistical Analyses

For all analyses, respective sample weights were applied to the data to adjust for nonresponse and the varying probabilities of selection, including those resulting from oversampling, yielding nationally representative findings. SUDAAN 10.0 [45], which accounts for the complex survey sample design, was used to generate prevalence estimates and 95% confidence intervals.

For NHIS and NSDUH, 2008 prevalence estimates were calculated, overall and by demographic subgroup, for current smoking and daily smoking among current smokers, and two sets of between-survey comparisons then made. The first comparison was made using the NHIS current smoking definition versus the NSDUH-S definition, and the second using the NHIS current smoking definition versus the NSDUH-M definition. To explore lifetime smoking of <100 cigarettes among current smokers, 2006–2008 NSDUH-S combined prevalence estimates were calculated, overall and by demographic subgroup. Two-sided *t*-tests were performed for both 2008 NHIS versus 2008 NSDUH comparisons to identify statistically significant differences at an alpha level of 0.05. Adjusted odds ratios with 95% confidence intervals were calculated for the 2006–2008 NSDUH-S combined analysis, controlling for age, gender, race/ethnicity, and education.

## 3. Results

### 3.1. Current Cigarette Smoking among Adults

Assessment of the NSDUH-S current smoking definition indicated that the overall prevalence (25.5%, 95%CI 24.7–26.2) was significantly higher than the NHIS overall prevalence (20.6%, 95%CI 19.9–21.4) (Table 1). This same pattern was observed for all subpopulations analyzed except the 50–64- and ≥65-year old age groups, Asians, and American Indians/Alaska Natives. Using the NSDUH-M current smoking definition, overall prevalence remained significantly higher (23.6%, 95%CI 22.8–24.3) than the NHIS overall prevalence. This same pattern was observed for the 18–25 and 26–34 years age groups, males, non-Hispanic whites, and college graduates.

### 3.2. Daily Cigarette Smoking among Current Smokers

Assessment of smoking frequency using the NSDUH-S current smoking definition indicated that the overall prevalence of daily smoking (63.3%, 95%CI 61.8–64.8) was significantly lower than the NHIS prevalence (79.7%, 95%CI 78.3–81.2) (Table 1). This same pattern was observed for all subpopulations analyzed except the ≥65 year old age group and American Indians/Alaska Natives. Using the NSDUH-M current smoking definition, the prevalence of daily cigarette smoking during the past 30 days remained significantly lower (68.2%, 95%CI 66.8–69.6) than the NHIS prevalence. This same pattern was observed for all subpopulations analyzed except the 26–34- and ≥65-year-old age groups, Hispanics or Latinos, Asians, and American Indians/Alaska Natives.

### 3.3. <100 Lifetime Cigarettes among Current Smokers

Among NSDUH-S current smokers, younger respondents had significantly greater odds of smoking fewer than 100 cigarettes during their lifetime (Table 2). Using persons aged ≥65 years as the referent, 18–24-year olds had 11.2 times greater odds (aOR, 95%CI: 4.8–26.1) and 25–34-year olds had 3.5 times greater odds (aOR, 95%CI: 1.5–8.7), of having a lifetime smoking level of <100 cigarettes. By gender, females had 1.2 times greater odds (aOR, 95%CI: 1.1–1.4) than males of having a lifetime smoking level <100 cigarettes. As compared to non-Hispanic whites, Hispanic or Latino smokers had 4.8 times greater odds (aOR, 95%CI: 4.2–5.5) of having a lifetime smoking level of <100 cigarettes, followed by American Indians/Alaska Natives (aOR, 95%CI: 3.6, 1.8–7.3), non-Hispanic blacks (aOR, 95%CI: 2.4, 2.0–2.8), and Asians (aOR, 95%CI: 2.2, 1.5–3.3). By education, smokers who graduated from college had 2.5 times greater odds (aOR, 95%CI: 1.9–3.2), and those with some college education had 1.7 times greater odds (aOR, 95%CI: 1.3–2.1), of having a lifetime smoking level of <100 cigarettes than those with less than a high school education.

## 4. Discussion

In comparisons between NHIS and NSDUH, NSDUH consistently yielded higher national overall and subpopulation estimates of current cigarette smoking among adults than NHIS and, among current smokers, lower estimates of daily smoking. However, with the use of the modified NSDUH-M current smoking variable definition that, like the NHIS definition, is restricted to respondents with lifetime cigarette use ≥100 cigarettes, estimates generally shifted closer to NHIS estimates, and several subgroups differences that were statistically significant for NHIS versus NSDUH-S became comparable for NHIS versus NSDUH-M. Specifically, estimate comparability occurred for the current smoking variable among 35–49-year olds, females, non-Hispanic black respondents, and those with <high school, high school graduate, or some college educational level, and, for the daily smoking variable, among 26–34 year olds and Asian respondents. Among Hispanic respondents, comparability occurred for both the current smoking variable and the daily smoking variable. In these instances, enough NSDUH respondents who reported smoking during the past 30 days had smoked *fewer than* 100 lifetime cigarettes (i.e., NSDUH-M) to negate the significant differences originally observed when level of lifetime cigarette use was not taken into account (i.e., NSDUH-S). The 100 cigarette prerequisite appeared to impact current smoking estimates much more extensively than it did smoking frequency estimates; that is, inclusion of the prerequisite produced comparability in estimates extensively across all four demographic categories for current smoking, whereas comparability occurred only minimally for daily smoking.

Subpopulations most impacted by the restriction of the current smoker variable definition to respondents with lifetime cigarette use ≥100 cigarettes appear to be younger adults and racial/ethnic minorities. The current smoking estimate comparability that occurred with use of the NSDUH-M current smoking definition represents a loss of significant differences originally observed between NHIS and NSDUH-S for the 35–49-years age group, females, non-Hispanic blacks, Hispanics, and the <high school, high school graduate, and some college educational levels. The daily smoking estimate comparability that occurred represents a loss of significant differences originally observed between NHIS and NSDUH-S for the 26–34-years age group, Asians, and Hispanics. Within this, Hispanic smoking prevalence appeared to be the most sensitive to differences in smoking variable definitions as this was the only group for which estimate comparability occurred across both current smoking and daily smoking.

These findings are consistent with other studies showing restriction of the adult current smoking definition to respondents with lifetime cigarette use ≥100 cigarettes leads to lower prevalence estimates [10, 12, 13], especially among minorities [46]. They are also consistent with previous studies that specifically found Hispanic smokers were most likely to be nondaily smokers and to smoke fewer days per month than non-Hispanic respondents [18, 19, 21–24, 31, 47]. It was the tobacco industry itself, however, that showed foresight into the relevance of such nuances and the subsequent opportunities afforded by what it termed “occasional smokers,” and during the 1990s took an interest in this group. Indeed, tobacco industry workshop materials from 1996 explained that occasional smokers may or may not self-identify as a smoker [47]. Data collection efforts by Philip Morris that took place in the late 1990s specifically focused on those who did *not* identify as a smoker and defined occasional smokers simply to be people who referred to themselves as nonsmokers, responded “yes” when asked if they smoked one or more cigarettes in the past year, and responded “no” when asked if they presently smoke at least a pack a week [48]. Internal communications summarizing the resulting data noted that “Hispanics represent substantially more than their fair share of occasional smokers” [49].

Husten (2009) [14] states that the stability of the behavior within any definitional category or categories of occasional use is an important consideration in determining a definition of the term. We take this line of thought a step further by applying stability criteria *within* a particular variable definition and *across* multiple subpopulations. The current analysis indicates that WHO's call for the provision of overall as well as demographic subpopulation data [6] may not be accurately met if a single current smoking definition is utilized for all subgroups when those same groups are known to differ on a key component of the variable's definition (i.e., occasional use). Like Husten, we reason that levels of consumption may be best left as continuous variables rather than presumptive cut-points, as there do not seem to be clear consumption levels that correlate with the onset of dependence or health risk. As noted, data that definitionally include rather than exclude lower consumption patterns have significant implications for the understanding of tobacco use and addiction and the development of prevention and cessation strategies—such as the extent to which intervention messages do versus do not address non-daily smoking [20], health risks of any smoking [31], motivations other than health effects [20], beliefs about ability to quit [23], situational triggers [31], social and cultural forces [23], and attitude changes [50]—especially for racial/ethnic minorities.

Measures relevant to occasional smokers are needed to be able to adequately monitor and describe their cigarette use, motivations, nicotine dependence, and cessation behaviors [50], underscoring the importance for national surveillance systems to use multiple comparable prevalence measures to capture diverse smoking behaviors, especially among subgroups. Consideration must be taken with regards, but not limited to, any screener questions, skip patterns, or closed data edits that result in a complete drop of certain respondents such that they are unable to be added back in when calculating prevalence estimates. An assumption of dropping respondents from certain questions is that the answers to these questions, had they been asked, would in most cases have been “no” or “not applicable” [15]. Much could thus be gained by maintaining one or two key smoking behavior questions across surveys, allowing researchers to retain rather than relinquish the ability to test this assumption [15] and subsequently capture, assess, and use these data to their fullest capacity. Further investigation of associations between the knowledge, attitudes, and behaviors of true never smokers (i.e., lifetime smoking level = 0) and graded levels of lifetime cigarette use >0 may provide additional help in determining whether a judicious cut-point exists for categorizing a respondent as an ever smoker versus a never smoker and, subsequently, in defining current smokers. In the meantime, investigators should use data most appropriate for addressing their specific research questions and subgroups of interest (e.g., relevant consumption levels, age group, racial/ethnic minority status, etc.).

### 4.1. Limitations

This paper has described how the use of a modified NSDUH current smoking variable definition that, like the NHIS definition, is restricted to respondents with lifetime cigarette use ≥100 cigarettes negates a notable number of significant differences among subpopulation otherwise observed between the two surveys. However, there are other central methodological differences in addition to question wording that were not assessed in the current analysis—such as survey mode, setting, context, and incentives—that may also contribute to discrepancies in current smoking estimates. In 1994, NSDUH changed from an interviewer administered survey mode for the tobacco questions to a self-administered survey mode for these questions. Findings from a random split sample conducted to measure the impact suggest that the self-administered mode may have resulted in higher reporting of current smoking behavior [51, 52]. NHIS tobacco questions, on the other hand, remain interviewer-administered. Further, NHIS interviews that either cannot be conducted or fully completed in person are administered by telephone, whereas NSDUH interview mode is strictly in person. In a study comparing telephone versus face-to-face interviewing of national probability samples, findings suggest telephone respondents to be more likely to present themselves in socially desirable ways than were face-to-face respondents [53]. More changes in the NSDUH mode of administration took place in 1999 when it shifted from paper and pencil interviews to ACASI. ACASI is thought to provide respondents with an enhanced sense of privacy, thus increasing their willingness to truthfully report their health behaviors. Indeed, a 2004 study comparing the 1999 and 2001 NSDUH and BRFSS prevalence estimates of adult binge drinking reported that—having ruled out other explanations such as differences in survey design, sampling, response rates and question wording—ACASI may have been responsible for the NSDUH estimates that were 2.4 to 9.2 percentage points higher than BRFSS estimates [54].

NHIS and NSDUH also differ in terms of overall survey context and question placement, which may influence respondents' perceptions of smoking itself [10]. NHIS primarily focuses on participants' health status with limited attention given to related licit substance use (cigarette and alcohol use), whereas NSDUH focuses almost entirely on substance-use behaviors, covering both licit and illicit substances, including marijuana, cocaine, crack, hallucinogens, inhalants, and nonmedical use of prescription drugs. In the NHIS context where cigarette use is one of the most serious health behaviors one can report respondents may perceive smoking to be one of the more undesirable behaviors they are being asked about, which may lead to underreporting [35, 55]. Conversely, in the NSDUH context respondents may perceive smoking to comparatively be one of the more socially acceptable behaviors they are being asked about and thus may be more comfortable acknowledging that they smoke [10].

In 2002, the NSDUH began paying respondents a $30 incentive upon completion of the survey, whereas the NHIS remains uncompensated. Although the results of a 2001 experiment indicated that the incentive would have no appreciable impact on prevalence estimates [56], “reality dictated otherwise” according to a SAMHSA report [57]. SAMHSA reports presenting NSDUH's summary of findings in 2001 and 2002 revealed increased prevalence estimates across the majority of substances queried in the survey [57], including cigarettes, alcohol, any illicit drug use, marijuana, and cocaine [58].

Lastly, in addition to survey mode, setting, context, and incentives, there are other factors that may affect prevalence estimates that also fell outside the scope of the current study, such as construct validity and differences in target populations, sampling methods, adjustments for non-response, and weighting. While all of the preceding may help explain observed differences in smoking prevalence estimates, more research in these areas is needed [10, 35].

## 5. Conclusions

Our study provides further information on how different smoking definitions between two national surveys may impact the overall and subpopulation prevalence estimates observed for some smoking behaviors. Our findings can be used to further inform tobacco control research and surveillance with regards to measurement of adult smoking behavior, including current use and frequency of use. Moreover, these findings may also inform how and why estimates differ by demographic subpopulation. Evidence-based, statewide tobacco control programs that are comprehensive, sustained, and accountable have been shown to reduce smoking rates, tobacco-related deaths, and diseases caused by smoking, with tobacco use monitoring critical to ensuring that program-related effects can be clearly measured [7]. Further research on methodological issues related to differing smoking prevalence estimates across tobacco control monitoring systems is needed, in particular to enhance the capacity of tobacco control surveillance to evaluate progress and further tobacco control efforts. Better understanding of why estimates may vary across data systems and among specific subpopulations, coupled with continued surveillance efforts, permits more accurate assessment of adult smoking prevalence and tobacco use behaviors.

## Figures and Tables

**Table 1:** Current cigarette smoking among adults^‡§¶^ and daily cigarette smoking among adults who currently smoke^∗∗††^ by demographic and current smoking variable definition—NHIS and NSDUH, 2008.

	Current cigarette smoking^‡§¶^									Daily cigarette smoking among current smokers^∗∗††^								
	NSDUH-S^‡^			NHIS^§^			NSDUH-M^¶^			NSDUH-S^‡^			NHIS^§^			NSDUH-M^¶^		
	%	LL	UL	%	LL	UL	%	LL	UL	%	LL	UL	%	LL	UL	%	LL	UL
Total	25.5*	24.7	26.2	20.6	19.9	21.4	23.6^†^	22.8	24.3	63.3*	61.8	64.8	79.7	78.3	81.2	68.2^†^	66.8	69.6

Demographic																		
Age																		
18–25 years	35.5*	34.6	36.5	21.4	19.4	23.5	28.2^†^	27.3	29.1	48.1*	46.4	49.8	74.3	70.0	78.7	59.9^†^	58.1	61.6
26–34 years	33.8*	32.1	35.5	25.2	23.4	27.1	31.5^†^	29.8	33.1	59.9*	56.7	63.2	72.6	69.2	76.1	64.3	61.2	67.4
35–49 years	27.6*	26.3	28.9	23.4	22.1	24.8	26.4	25.2	27.7	66.9*	64.4	69.4	83.1	80.9	85.4	69.7^†^	67.2	72.1
50–64 years	22.1	20.2	23.9	21.5	20.2	22.9	21.7	19.8	23.5	72.1*	68.4	75.7	83.6	81.2	86.1	73.3^†^	69.6	77.0
≥65 years	9.9	8.2	11.7	9.3	8.2	10.4	9.7	7.9	11.4	76.3	68.7	84.0	81.6	77.3	85.9	78.2	71.2	85.3
Gender																		
Male	28.5*	27.4	29.6	23.1	22.0	24.2	26.4^†^	25.3	27.5	62.2*	60.1	64.2	77.4	75.2	79.6	66.9^†^	65.0	68.9
Female	22.7*	21.7	23.6	18.3	17.3	19.3	20.9	20.0	21.8	64.7*	62.4	66.9	82.5	80.7	84.3	69.7^†^	67.4	72.1
Race/ethnicity																		
White non-Hispanic	26.5*	25.6	27.4	22.0	21.1	23.0	25.1^†^	24.2	25.9	69.0*	67.2	70.8	83.4	81.8	85.0	72.8^†^	71.1	74.5
Black non-Hispanic	27.2*	25.0	29.4	21.2	19.4	23.1	25.0	22.9	27.2	54.7*	49.6	59.7	76.2	72.4	80.0	58.6^†^	53.3	63.9
Hispanic or Latino	21.4*	19.6	23.3	15.8	14.2	17.4	17.3	15.6	19.0	39.0*	34.8	43.1	59.2	53.6	64.8	47.7	42.8	52.6
Asian^‡‡^	12.5	9.5	15.6	9.8	7.5	12.1	10.6	7.7	13.5	51.5*	40.3	62.8	79.0	71.6	86.3	60.8	49.2	72.4
American Indian/Alaska Native^§§^	47.2	35.5	58.9	32.4	23.8	41.1	42.7	30.5	54.8	59.2	39.5	78.9	69.2	49.8	88.6	65.1	45.8	84.3
Education^¶¶^																		
<High school	35.0*	32.8	37.1	27.6	25.6	29.5	32.6	30.5	34.8	68.3*	64.5	72.1	83.8	80.9	86.8	72.8^†^	69.3	76.3
High school graduate	29.7*	28.3	31.1	25.3	23.8	26.8	28.2	26.9	29.6	71.0*	68.6	73.4	83.6	80.8	86.3	74.4^†^	72.1	76.8
Some college	27.1*	25.6	28.5	22.7	21.3	24.1	24.8	23.4	26.2	61.2*	58.0	64.3	79.9	77.2	82.6	66.6^†^	63.6	69.5
College graduate	14.0*	12.9	15.2	8.9	8.0	9.8	12.9^†^	11.7	14.0	47.4*	43.0	51.8	67.1	61.9	72.3	51.8^†^	47.1	56.4

*Significant difference between NHIS and NSDUH-S, *P* < .05.

^†^Significant difference between NHIS and NSDUH-M, *P* < .05.

^‡^NSDUH respondents ≥18 years of age who reported smoking part or all of a cigarette during the preceding 30 days.

^§^NHIS respondents ≥18 years of age who have smoked ≥100 cigarettes in their lifetime and reported they now smoke cigarettes either every day or some days.

^¶^NSDUH respondents ≥18 years of age who have smoked ≥100 cigarettes in their lifetime and reported smoking part or all of a cigarette during the preceding 30 days.

**NSDUH current cigarette smokers ≥18 years of age who reported smoking on all of the preceding 30 days.

^††^NHIS current cigarette smokers ≥18 years of age who reported they now smoke cigarettes every day.

^‡‡^Non-Hispanic, and does not include Native Hawaiian and Other Pacific Islander.

^§§^Non-Hispanic. Wide variances in estimates reflect small sample sizes.

^¶¶^Among respondents ≥26 years of age.

**Table 2:** Level of lifetime cigarette use* <100 cigarettes among adults who currently smoke cigarettes^†^, by demographic—NSDUH 2006–2008.

	Level of lifetime smoking <100 cigarettes among current smokers					
	Prevalence estimates			Adjusted odds ratios^‡^		
	%	LL	UL	aOR	LL	UL
Total	7.1	6.7	7.4			

Demographic						
Age						
18–25 years	19.1	18.3	19.8	11.2	4.8	26.1
26–34 years	6.9	6.1	7.8	3.5	1.5	8.7
35–49 years	3.8	3.1	4.4	2.0	0.9	4.8
50–64 years	1.8	1.2	2.5	1.1	0.4	2.7
≥65 years	1.6	0.3	2.9	1.0	1.0	1.0
Gender						
Male	6.9	6.4	7.4	1.0	1.0	1.0
Female	7.3	6.8	7.8	1.2	1.1	1.4
Race/Ethnicity						
White non-Hispanic	5.0	4.6	5.3	1.0	1.0	1.0
Black non-Hispanic	8.6	7.5	9.7	2.4	2.0	2.8
Hispanic or Latino	17.1	15.3	18.9	4.8	4.2	5.5
Asian^§^	12.5	8.8	16.2	2.2	1.5	3.3
American Indian/Alaska Native^¶^	11.8	6.8	16.9	3.6	1.8	7.3
Education**						
<High school	5.5	4.6	6.4	1.0	1.0	1.0
High school graduate	5.0	4.5	5.5	1.1	0.8	1.4
Some college	7.8	7.2	8.5	1.7	1.3	2.1
College graduate	8.3	7.2	9.4	2.5	1.9	3.2

*Among NSDUH respondents ≥18 years of age who reported ever smoking part or all of a cigarette, those who have smoked ≥100 cigarettes in their lifetime versus those who have smoked <100.

^†^NSDUH respondents ≥18 years of age who reported smoking part or all of a cigarette during the preceding 30 days.

^‡^Adjusted for age, gender, race/ethnicity, and education.

^§^Non-Hispanic, and does not include Native Hawaiian and Other Pacific Islander.

^¶^Non-Hispanic. Wide variances in estimates reflect small sample sizes.

**Among respondents ≥26 years of age.
